# Human embryonic stem cells contribute to embryonic and extraembryonic lineages in mouse embryos upon inhibition of apoptosis

**DOI:** 10.1038/cr.2017.138

**Published:** 2017-11-03

**Authors:** Xuepeng Wang, Tianda Li, Tongtong Cui, Dawei Yu, Chao Liu, Liyuan Jiang, Guihai Feng, Lei Wang, Rui Fu, Xinxin Zhang, Jie Hao, Yukai Wang, Liu Wang, Qi Zhou, Wei Li, Baoyang Hu

**Affiliations:** 1 State Key Laboratory of Stem Cell and Reproductive Biology, Institute of Zoology, Chinese Academy of Sciences, Beijing 100101, China;; 2 University of Chinese Academy of Sciences, Beijing 100049, China;; 3 College of Life Science, Northeast Agricultural University of China, Harbin, Heilongjiang 150030, China

## Dear Editor,

Recently, interspecies chimera formation has been established in rodents by injection of rat pluripotent stem cells (PSCs) into mouse early embryos, and such a system provides an *in vivo* assay to test the developmental potential of human PSCs (hPSCs)^1^. In addition, the interspecies chimeras formed between hPSCs and large animal embryos would open new avenues to generate human tissues and organs for regenerative medicine^2^. In rodents, embryonic stem cells (ESCs) and epiblast stem cells (EpiSCs) have been derived from inner cell mass (ICM) of the blastocyst and post-implantation epiblast respectively^3,4^. Although both being pluripotent, the ESCs and EpiSCs are considered to represent two distinct states of pluripotency, the naïve and primed pluripotent state. Only naïve state ESCs can colonize the early embryos before the blastocyst stage to form adult chimeras and transmit to the germline, while the primed state EpiSCs can only integrate into the post-implantation embryos^5,6^. Intriguingly, although derived from the ICM, the human ESCs (hESCs) are similar to the mouse EpiSCs in many aspects such as the morphology and self-renewal pathways, hence they are considered to represent the primed pluripotent state. Several studies have reported the generation of human-animal interspecies chimeras using the stage-matching hESCs. For example, primed hPSCs were engrafted into *in vitro* cultured gastrula-stage mouse embryos to form chimeras, and the primed hPSCs were also converted into a naïve-like state and then were injected into pre-implantation embryos to form mouse or pig chimeras^7,8,9^. Recently, a non-stage-matching approach has been established to generate mouse-rat interspecies chimeras by inhibiting apoptosis of the primed ESCs^10^. We hence hypothesize that inhibition of apoptosis may enable the primed hESCs to form interspecies chimeras upon injection into mouse pre-implantation stage embryos.

**Figure 1:**
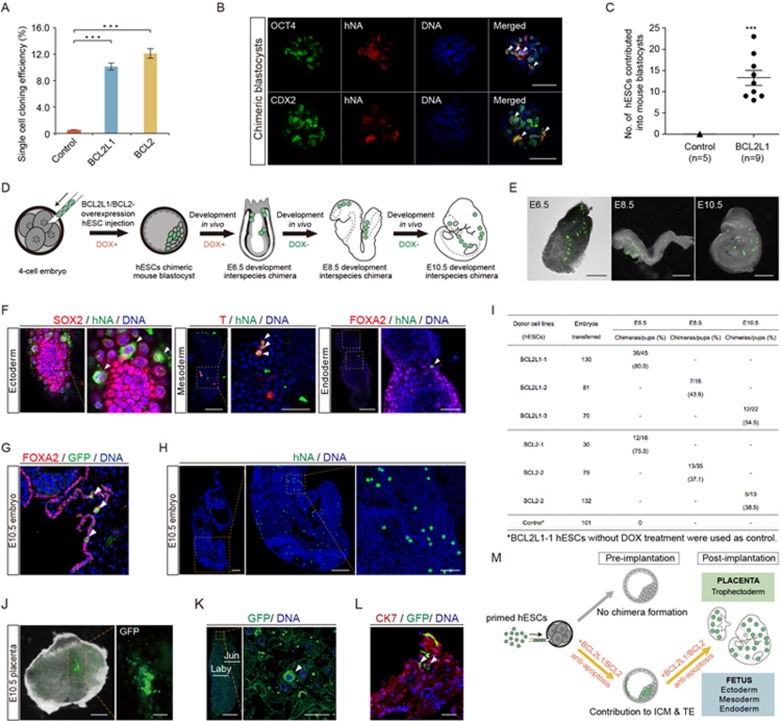
Overexpression of anti-apoptotic genes enables hESCs to differentiate into embryonic and extraembryonic lineages in pre- and post-implantation mouse embryos. **(A)** Colony formation efficiency of the *BCL2L1*- and *BCL2*-overexpressing hESCs. The wild-type hESCs were used as control. Data are displayed as mean ± SEM of three biological replicates. ^***^P < 0.001. **(B)** Immunofluorescent staining of OCT4 (specific marker of the ICM lineage), CDX2 (specific marker of the TE lineage) and the human nuclear antigen (hNA) in chimeric blastocysts of *BCL2L1*-overexpressing hESCs and mouse early embryos. White arrowheads indicate OCT4 and hNA double-positive cells, and CDX2 and hNA double-positive cells respectively. Scale bars, 100 μm. **(C)** Quantitative analysis of the number of hNA-positive hESC-derived cells integrated into interspecies chimeric mouse blastocysts. *BCL2L1* transgenic hESCs without DOX treatment were used as control. Data are displayed as mean ± SEM of three biological replicates. ^***^P < 0.001. **(D)** Schematic overview of the strategy to generate post-implantation interspecies chimeras by injecting *BCL2L1*/*BCL2*-overexpressing hESCs into 4-cell stage mouse embryos. **(E)** Representative phase contrast images of E6.5, E8.5 and E10.5 mouse chimeric embryos containing GFP-positive hESC-derived cells. Scale bars, 150 μm (left), 200 μm (middle), 1 mm (right). **(F)** Immunofluorescent staining of ectoderm-specific marker SOX2 (left panel), mesoderm-specific marker T (Brachyury) (middle panel), and endoderm-specific marker FOXA2 (right panel) in human-mouse interspecies chimeric embryos at E6.5. hNA was used for detecting hESC-derived cells. Scale bars, 100 μm (left) and 50 μm (right) for each panel. **(G)** Immunofluorescent staining of FOXA2 in E10.5 interspecies chimeric embryos. hNA staining was used for detecting hESC-derived cells. White arrowheads indicate FOXA2 and GFP double-positive cells. **(H)** Immunofluorescent staining of hNA for detection of *BCL2L1*-overexpressing hESC-derived cells in E10.5 interspecies chimeric embryos. Scale bars, 100 μm (left) and 50 μm (middle and right). **(I)** Summary of chimera formation efficiencies of the *BCL2L1*- and *BCL2*-overxpressing hESCs in post-implantation mouse embryos. BCL2L1-1 hESCs without DOX treatment were used as control. **(J)** Representative phase contrast image of E10.5 interspecies chimeric placenta containing hESC-derived GFP-positive cells. Scale bars, 500 μm and 200 μm. **(K)** Immunofluorescent staining of GFP for detection of *BCL2L1*-overexpressing hESC-derived cells in E10.5 interspecies chimeric placenta. White arrowheads indicate derivative cells of hESCs. Jun, Junctional zone. Laby, Labyrinth zone. Scale bars, 500 μm (left) and 50 μm (right). **(L)** Immunofluorescent staining of trophoblast lineage marker CK7 (Cytokeratin 7) and GFP in E10.5 interspecies chimeric placenta. White arrowheads indicate CK7 and GFP double-positive cells. Scale bar, 50 μm. **(M)** Illustrative model showing that inhibition of apoptosis promotes primed hESCs to form interspecies chimeras in mouse embryos.

We used a doxycycline (DOX)-inducible system for transient induction of human anti-apoptotic genes, *BCL2L1* or *BCL2*, together with the *DsRed* reporter gene, in hESCs carrying a constitutively expressed reporter gene *GFP* (Supplementary information, Figure S1A and S1B). The *BCL2L1*- and *BCL2*-overexpressing hESCs maintained their pluripotent state and could differentiate into embryoid bodies (EBs) expressing marker genes of all three germ layers (Supplementary information, Figure S1C-S1F). Compared with the control hESCs, the *BCL2L1*- and *BCL2*-overexpressing hESCs displayed much lower levels of spontaneous apoptosis and significantly higher single cell cloning efficiency (Supplementary information, Figure S1G and Figure 1A). Global gene expression profiles revealed that *BCL2L1* and *BCL2* overexpression had little effect on the hESC pluripotent state, as only 40 and 48 differentially expressed genes (DEGs) were identified in the *BCL2L1*- and *BCL2*-overexpressing hESCs respectively, which were involved in functions related to cellular response to DNA damage stimuli and extracellular stimuli, and regulation of apoptotic process (Supplementary information, Figure S1H-S1K). Consistently, *BCL2L1*- and *BCL2*-overexpressing hESCs were clustered with the primed state hESCs but not with the previously reported naïve state hESCs by principal component analysis^9^, and did not upregulate naïve marker gene expression (Supplementary information, Figure S1L and S1M). Taken together, these results suggest that *BCL2L1* or *BCL2* overexpression can inhibit apoptosis in hESCs without affecting their pluripotent state.

Next, we investigated whether the inhibition of apoptosis could enable the hESCs to colonize early stage mouse embryos. A total of 76 blastocysts were generated from 89 4-cell embryos injected with *BCL2L1*-overexpressing hESCs, and 72 of them (∼95%) contained GFP-positive human cells. In contrast, GFP fluorescence was scarcely observed in the blastocysts injected with control hESCs due to apoptosis (Supplementary information, Figure S2A, S2B and Table S1, S2). Intriguingly, the hESCs contributed to both the ICM and trophectoderm (TE) of the chimeric blastocysts as confirmed by the GFP fluorescence and immunofluorescence staining against lineage-specific markers and the human nuclear antigen (hNA) in the chimeric blastocysts (Figure 1B and Supplementary information, Figure S2A). Statistical analysis showed that on average 13 hNA-positive cells were present in each chimeric blastocyst (Figure 1C).

We next investigated the contribution of the *BCL2L1*- and *BCL2*-overexpressing hESCs in post-implantation mouse embryos (Figure 1D). Clear GFP signals were observed in the mouse embryos dissected at embryonic day 6.5 (E6.5), E8.5 and E10.5, indicating the presence of hESC-derived cells (Figure 1E). PCR analysis also detected the existence of the hESC-carried transgene in 5/10 dissected E6.5 embryos (Supplementary information, Figure S2C). Moreover, immunostaining of lineage-specific markers showed that the hESCs could differentiate into all three germ layers in E6.5 chimeric mouse embryos (Figure 1F). Analysis of E10.5 chimeric embryos also showed the differentiation of hESCs into FOXA2-expressing endoderm cells (Figure 1G). Of embryos dissected at E6.5, E8.5 and E10.5, around 80% (36 out of 45 fetuses), 44% (7 out of 16 fetuses) and 55% (12 out of 22 fetuses) contained GFP-positive human cells from the *BCL2L1*-overexpressing hESC injection group (Figure 1E and 1I). We next analyzed the proportions of human cells in chimeric embryos. 10 E8.5 chimeric embryos were analyzed using fluorescence-activated cell sorting (FACS), and about 0.7% of total cells were GFP-positive (Supplementary information, Figure S2D). Immunostaining of the tissue sections of 5 E10.5 chimeric embryos, and a sensitive PCR assay to quantify the human mitochondrial DNA ratio at the whole-embryo level in 10 independent E10.5 chimeric embryos, both confirmed that the hESCs could contribute up to 1% of total cells in the post-implantation chimeric embryos (Figure 1H and Supplementary information, Figure S2E and S2F).

Intriguingly, the presence of GFP signals could also be observed in the extraembryonic tissues such as the placenta and yolk sac at E10.5 (Figure 1J; Supplementary information, Figure S2G). The immunostaining assay further confirmed the incorporation of GFP-expressing cells into the junctional zone of E10.5 chimeric placenta (Figure 1K). Notably, the co-localization of GFP signal and CK7, a trophoblast marker, was also detected (Figure 1L). Moreover, PCR analysis validated the presence of the *BCL2L1* transgene in 6 independent E10.5 chimeric placenta samples (Supplementary information, Figure S2H). Taken together, these results demonstrate that the *BCL2L1*- and *BCL2*-overexpressing hESCs could differentiate toward the extraembryonic lineages in post-implantation mouse embryos.

In summary, we demonstrate here that inhibition of apoptosis of hESCs by overexpression of *BCL2L1* and *BCL2* enabled the hESCs to efficiently form interspecies human-mouse chimeras upon injection into 4-cell mouse embryos without affecting their pluripotent state (Figure 1M). This non-stage-matching approach for human-animal chimera production opens a new avenue to generate human organs in organ-deficient large animals. Moreover, the finding that the primed state hESCs could differentiate into extraembryonic lineages *in vivo* in mouse embryos, may shed light on the developmental potency of hESCs and the differences in pluripotency maintenance and regulation across species.
