# Supplementary material for: Human embryonic stem cells contribute to embryonic and extraembryonic lineages in mouse embryos upon inhibition of apoptosis
Source: Cell Res. 2017 Nov 3;28(1):126–9. doi: 10.1038/cr.2017.138 (PMC5752840; doi:10.1038/cr.2017.138)
Supplement: Supplementary information, Figures and Methods — Supplementary information, Figures S1–S2 [file cr2017138x1.pdf]

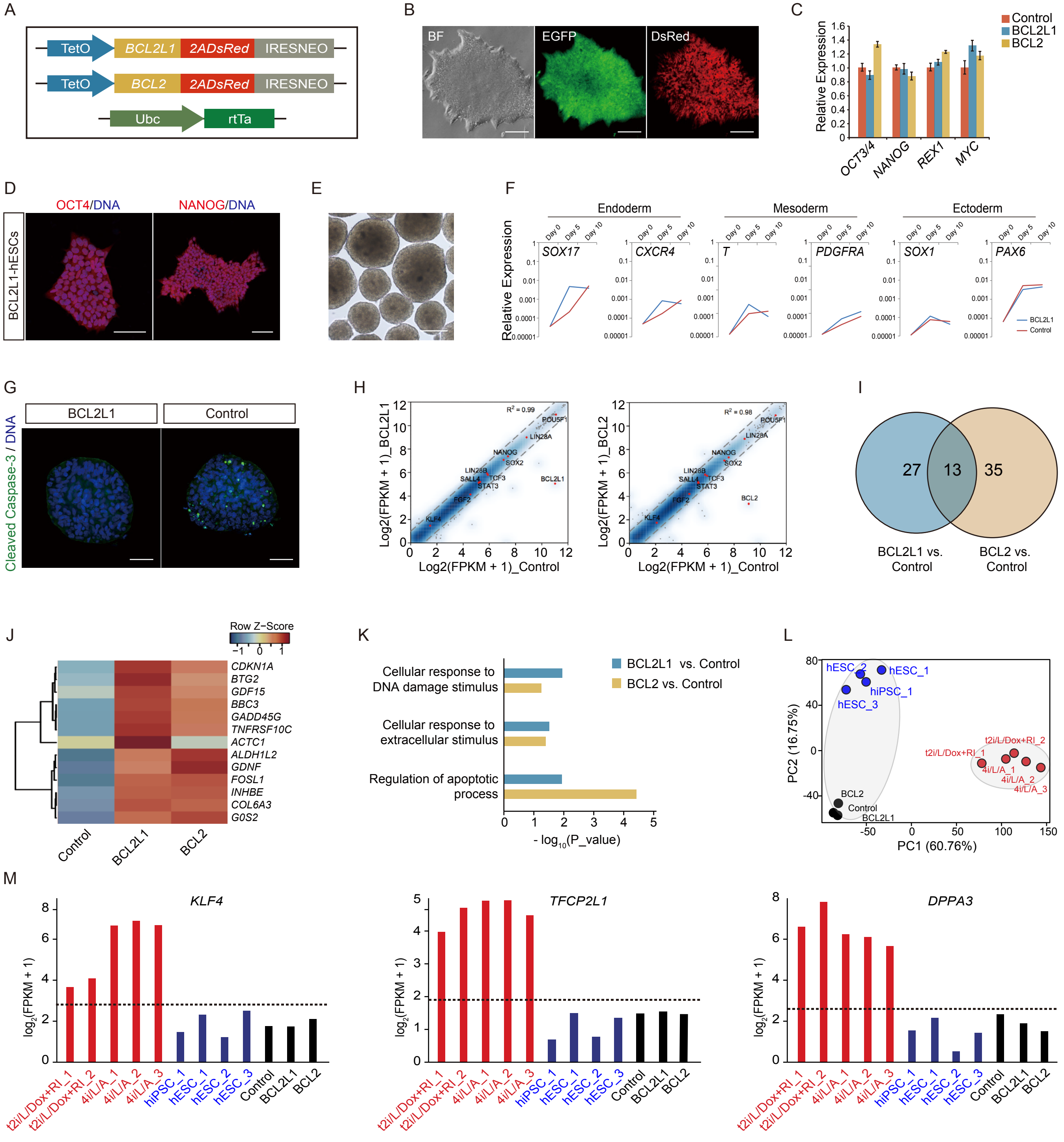

A
TetO
BCL2L1
2ADsRed
IRESNEO
TetO
BCL2
2ADsRed
IRESNEO
Ubc
rtTa
B
BF
EGFP
DsRed
C
Control
BCL2L1
BCL2
Relative Expression
OCT3/4
NANOG
REX1
MYC
D
OCT4/DNA
NANOG/DNA
BCL2L1-hESCs
E
F
Endoderm
Mesoderm
Ectoderm
Relative Expression
Day 0
Day 5
Day 10
SOX17
CXCR4
T
PDGFRA
SOX1
PAX6
BCL2L1
Control
G
BCL2L1
Control
Cleaved Caspase-3 / DNA
H
Log2(FPKM + 1)_BCL2L1
Log2(FPKM + 1)_Control
R² = 0.99
POU5F1
LIN28A
NANOG
SOX2
LIN28B
SALL4
TCF3
STAT3
FGF2
KLF4
BCL2L1
Log2(FPKM + 1)_BCL2
R² = 0.98
BCL2
I
27
13
35
BCL2L1 vs. Control
BCL2 vs. Control
J
Row Z−Score
CDKN1A
BTG2
GDF15
BBC3
GADD45G
TNFRSF10C
ACTC1
ALDH1L2
GDNF
FOSL1
INHBE
COL6A3
G0S2
Control
BCL2L1
BCL2
K
Cellular response to DNA damage stimulus
Cellular response to extracellular stimulus
Regulation of apoptotic process
BCL2L1 vs. Control
BCL2 vs. Control
- log10(P_value)
L
hESC_2
hESC_1
hiPSC_1
hESC_3
t2i/L/Dox+RI_2
t2i/L/Dox+RI_1
4i/L/A_1
4i/L/A_2
4i/L/A_3
BCL2
Control
BCL2L1
PC2 (16.75%)
PC1 (60.76%)
M
KLF4
TFCP2L1
DPPA3
log2(FPKM + 1)
t2i/L/Dox+RI_1
t2i/L/Dox+RI_2
4i/L/A_1
4i/L/A_2
4i/L/A_3
hiPSC_1
hESC_1
hESC_2
hESC_3
Control
BCL2L1
BCL2

Supplementary Figure S1

Derivation and characterization of BCL2L1-/BCL2-overexpressing hESCs.

(A) Schematic overview of plasmid constructs for doxycycline (DOX)-inducible overexpression of human *BCL2L1* and *BCL2* transgenes. **(B)** Representative image of a hESC colony simultaneously expressing EGFP and DsRed by DOX treatment, under bright field (BF) and dark field. Scale bars, 200 μm. **(C)** Quantitative real-time PCR analysis of expression levels of pluripotency marker genes in BCL2L1- and BCL2-overexpressing hESCs (represented as BCL2L1 and BCL2 respectively). The wild-type hESCs were used as control. Data are displayed as mean ± SEM of three biological replicates. **(D)** BCL2L1-overexpressing hESCs showed positive immunofluorescent staining for pluripotency markers OCT4 and NANOG. Scale bars, 100 μm. **(E)** Morphology of EBs formed from BCL2L1-overexpressing hESCs. Scale bar, 200 mm. **(F)** Quantitative real-time PCR analysis of expression levels of the differentiation markers, *SOX17*, *CXCR4*, *T*, *PDGFRA*, *SOX1* and *PAX6* in EBs derived from the BCL2L1-overexpressing hESCs (red) and wild-type hESCs (blue). **(G)** Immunofluorescent staining of the apoptotic marker Cleaved Caspase-3 in BCL2L1-overexpressing and wild-type hESCs. Note that extensive apoptosis was detected in wild-type hESCs but not the BCL2L1-overexpressing hESCs. Scale bars, 100 μm. **(H)** Scatter plot comparison of transcriptomes of BCL2L1-/BCL2-overexpressing hESCs and control hESCs. The raw FPKM (fragments per kilobase of exon per million fragments mapped) value for each gene was transformed to log2 value, and genes with more than 1 FPKM in each sample were shown. The pluripotent genes, *BCL2L1* and *BCL2* were labeled in the diagram. Dashed lines depict the 2-fold changes. The R2 was determined by Pearson's correlation. The wild-type hESCs were used as control. **(I)** Venn diagram showing the differentially expressed genes (DEGs) between BCL2L1-overexpressing and wild-type hESCs (BCL2L1 vs WT), and DEGs between BCL2-overexpressing and wild-type hESCs (BCL2 vs WT). The threshold was set as p value ≤ 0.05 and fold change ≥ 2. 13 genes overlapped between two groups were listed in Fig S1E. **(J)** The differentially expressed genes which were highly expressed both in BCL2L1- and BCL2-overexpressing hESCs compared to wild-type hESCs.

(K) Gene ontology (GO) analysis of differentially expressed genes (DEGs) between BCL2L1-overexpressing and wild-type hESCs (BCL2L1 vs. Control), and DEGs between BCL2-overexpressing and wild-type hESCs (BCL2 vs. Control). **(L)** Principle component analysis of the transcriptome of each type hESCssp according to the expression level of RefSeq gene. The Principle component 1 (PC1) contributed to 60.76% variance among samples and split all samples into two groups which marked by ellipses. t2i/L/Dox+RI_1, t2i/L/Dox+RI_2, 4i/L/A_1, 4i/L/A_2 and 4i/L/A_3 are putative naïve state human ESCs. hiPSC_1, hESC_1, hESC_2 and hESC_3 are the prime state hESC control. **(M)** Gene expression levels of naïve pluripotency state marker genes in each cell type.

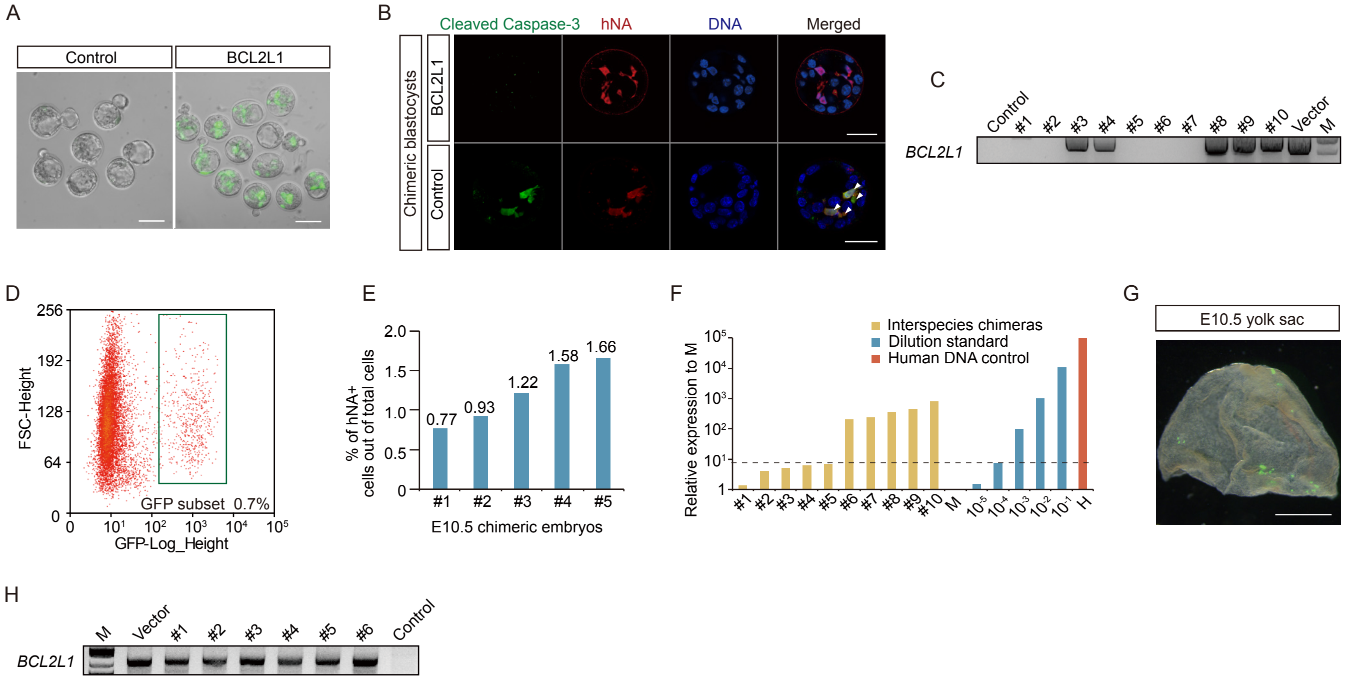

A
Control
BCL2L1
B
Cleaved Caspase-3
hNA
DNA
Merged
Chimeric blastocysts
BCL2L1
Control
C
Control
#1
#2
#3
#4
#5
#6
#7
#8
#9
#10
Vector
M
BCL2L1
D
FSC-Height
GFP-Log_Height
GFP subset 0.7%
E
% of hNA+ cells out of total cells
0.77
0.93
1.22
1.58
1.66
#1
#2
#3
#4
#5
E10.5 chimeric embryos
F
Interspecies chimeras
Dilution standard
Human DNA control
Relative expression to M
#1 #2 #3 #4 #5 #6 #7 #8 #9 #10 M 10^{-5} 10^{-4} 10^{-3} 10^{-2} 10^{-1} H
G
E10.5 yolk sac
H
M
Vector
#1
#2
#3
#4
#5
#6
Control
BCL2L1

Supplementary Figure S2

Identification of the chimeric ability of BCL2L1-/BCL2-overexpressing hESCs.

(A) Representative phase contrast images showing GFP signals in blastocysts injected with control hESCs and BCL2L1-overexpressing hESCs at the 4-cell stage. *BCL2L1* transgenic hESCs without DOX treatment were used as control. Scale bars, 100 μm. **(B)** Immunofluorescent staining of the apoptotic marker Cleaved Caspase-3 in mouse blastocysts which were injected with BCL2L1-overexpressing and control hESCs respectively at 4-cell stage. White arrowheads indicate the detected apoptotic cells. *BCL2L1* transgenic hESCs without DOX treatment were used as control. Scale bars, 50 μm. **(C)** PCR analysis of the presence of BCL2L1 transgene showing the contribution of BCL2L1-overexpressing hESCs in E6.5 chimeric embryos. M, marker. **(D)** Fluorescence activated cell sorting (FACS) analysis of the incorporation ratio (~0.7%) of GFP-positive hESC-derived cells in 10 pre-mixed E8.5 chimeric mouse embryos. **(E)** Histogram showing ratio of hNA-positive cells out of total cells per cross section in 5 individual E10.5 interspecies chimeric embryos. **(F)** Quantitative PCR analysis for human mitochondrial DNA indicated the presence of human cells in mouse embryos at E10.5 following injection of BCL2L1-overexpressing hESCs. A human mitochondrial DNA control (red bar) and a series of human-mouse cell dilutions (blue bars) were run in parallel to estimate the degree of human cell contribution in interspecies chimeras (yellow bars). The dashed line indicates the detection level of human mitochondrial DNA equivalent to a dilution of 1 human cell in 10,000 mouse cells. **(G)** Representative phase contrast image of E10.5 interspecies chimeric yolk sac containing hESC-derived GFP-positive cells. Scale bars, 500 μm. **(H)** Genomic PCR analysis of chimeric E10.5 placenta derived from hESCs mouse embryos injection. Primers targeting *BCL2L1* were used to detect the contribution of BCL2L1-overexpressing hESCs in chimeric placenta. M, marker.

Materials and Methods

Mice and ethical review

Mice were purchased from Beijing Vital River Company. All animal experiments were performed in compliance with the guidelines of the Institute of Zoology, Chinese Academy of Sciences. The cross-species chimeric experiments performed were reviewed and approved by the Ethics Committee of Institute of Zoology, Chinese Academy of Sciences, and followed the ethical guidelines for human embryonic stem cell research released by the International Society for Stem Cell Research (ISSCR).

Vector construction and virus package

For *BCL2L1-2AdsRed-IRESNEO* and *BCL2-2AdsRed-IRESNEO* vector construction, the *BCL2L1* and *BCL2* fragments were cloned from human cDNA library. 2AdsRed-IRESNEO fragments were cloned from PB-2AdsRed-IRESNEO vector and ligated with *BCL2L1* or *BCL2* fragment by use In-fusion cloning kit (Clontech). *BCL2L1-2AdsRed-IRESNEO* and *BCL2-2AdsRed-IRESNEO* were integrated into FUW-tet-ON vector by use In-fusion cloning kit (Clontech) in the final. For virus package, PMD2G, PSPAX and BCL2L1-2AdsRed-IRESNEO or BCL2-2AdsRed-IRESNEO vector were transfected into 293T cells by use lipofectamine3000 (ThermoFisher). Viral supernatant was collected 36-48h post transfection.

Derivation and cultivation of hESCs

All the reagents and media used for this study were purchased from Life Technologies Inc. unless otherwise mentioned. hESCs were derived from human blastocysts as previously reported. Established prime state hESCs and BCL2L1/BCL2-overexpression hESC lines were cultured with E8 medium on matrigel coated 6-well dishes. All of the stem cells were cultured at 37°C in a 5% CO_2 incubator. Medium were changed every other day.

Derivation of AAVS1-Knockin cell line

Well cultured prime state hESCs were digested by accutase and collected by

centrifuge. 10^6 hESCs were electroporated with 10 μg AAVS1-CAG-EGFP vector and 10 μg sgRNA-CAS9 expression vector by use 4D-Nucleofector (Lonza) using P3 Primary Cell 4D-Nucleofector kit (Lonza). Transfected cells were seeded onto mitotically inactivated MEFs in E8 medium combined with Revita-cell supplement (ThermoFisher). In the next day, medium was changed into standerd hESCs culture medium as previously reported. Three days later, 0.5 μg/ml puro was added in culture medium and after selection for 2 weeks, GFP-positive and puro-resistant clones were picked and identified by PCR.

Embryoid body formation

For EBs formation, prime state hESCs were digested into single cells by accutase and were transferred to low-cell binding U-bottom 96-well plate (NUNC) in the medium containing GMEM (Sigma), 15% KnockOut serum replacement, 0.1 mM Glutamine, 0.1 mM NEAA, 0.1 mM b-mercaptoethanol. The medium was changed every other day. After 5 days and 10 days, the EBs were collected and stored in Trizol reagent in -80°C. RT-PCR was performed to analyze specific gene expression for the three embryonic germ layers-endoderm (SOX17, CXCR4), mesoderm (T, PDGFA), and ectoderm (SOX1, PAX6).

Real-time PCR

Total RNA was isolated from freshly obtained cells using Trizol reagent. First strand cDNA was synthesized using MMLV reverse transcriptase (Promega) and oligo-dT (Promega) according to the manufacturer’s instructions. Real-time PCR was performed using SYBR Green Real-time PCR Master Mix (Toyobo) on Agilent Mx3005P. All primers used in the research are listed in Table S2.

Interspecies chimera generation

Mouse embryos were used as recipients for interspecies chimera generation. Mouse 2-cell stage embryos were recovered from 1.5 days post coitus (d.p.c.) oviducts and cultured to 4-cell stage embryos with KOSM medium (Sigma). For interspecies

chimera generation, anti-apoptotic gene transgenic hESCs were treated with or without 2 μg/ml DOX 24 hours before injection. Anti-apoptotic gene transgenic hESCs were digested into single cells using Accutase and suspended in culture medium. Approximately 10 BCL2L1-/BCL2-overexpression hESCs were microinjected into mouse 4-cell stage embryos or blastocysts to produce interspecies chimeric embryos. Injections were performed with a micromanipulation system. After being cultured for about 24 h in KSOM medium (Sigma) with or without DOX treatment (2 μg/ml, Sigma), these manipulated 4-cell staged embryos developed to blastocysts. Chimeric blastocysts were transferred into the uteri of 2.5 d.p.c. pseudopregnant CD-1 mice. Surrogate mothers were fed with DOX containing water (2 mg/ml) until the embryos developed to E6.5. The embryos or placentas were analyzed for EGFP expression, real-time PCR, immunofluorescence staining and fluorescence-activated cell sorting at E6.5, E8.5 and E10.5.

Immunofluorescence staining

Stained cells and embryos were fixed with 4% paraformaldehyde and then permeabilized with 0.5% Triton X-100 in PBS for 10 min followed by blocking with 2% BSA (Sigma) in PBS. The cells were incubated with primary antibodies overnight at 4°C, followed by the use of secondary antibodies (Jackson Immuno Research) at room temperature for 1h. Finally, DNA was stained with 10 μg/ml Hoechst 33342 for 30 min. E10.5 embryos and placentas were embedded in OCT (VWR), frozen and cryosectioned at 10 μm, then stained as described above. Images were captured with a Zeiss LSM780 Meta inverted confocal microscope. Antibodies against OCT4 (Santa Cruz), NANOG (Millipore), CDX2 (Cell signaling), Cleaved-Caspase 3 (Cell signaling), hNA (Millipore and Abcam), GFP (Abcam), Foxa2 (Santa Cruz), T (Santa Cruz), CK7 (Abcam) were used. Digital images of fluorescent signals were processed using Imaris 8.0.2, cropped and resized using Adobe Photoshop. Cell counting was achieved by Imaris 8.0.2. Composite panels were generated using Adobe Illustrator CC.

RNA-seq library preparation and data processing

Total RNA was extracted from cultured cells by Trizol reagent (Invitrogen). For RNA-seq library construction, The PolyA+ tailed RNA purification were performed for each sample using MicroElute RNA Clean Up Kit (OMEGA). Sequencing was performed on an Illumina HiSeq 4000 sequencer with 150 bp paired-end sequencing reactions. The RNA-seq reads of each sample were mapped to the human hg19 genome assembly independently by the HISAT2 software using the annotated gene structures as templates. Default parameters of HISAT2 were used except with the option "--dta-cufflinks" opening. Reads with unique genome location were reserved for gene expression calculation using Cufflinks (version 2.0.2) with the option "--GTF". Point plots were produced by the smooth scatter functions of R. And the Pearson correlation coefficients were showed in the figures. The heatmaps were produced by the heatmap.2 function of R. Gene ontology analysis of differentially expressed genes were analyzed in DAVID and processes were selected based on p values smaller than 0.05.

The accession number for the RNA-seq expression values reported in this paper is GSE100862.

Statistical analysis

All data are presented as mean ± SEM. Each experiment included at least three independent samples. Statistical parameters including statistical analysis, statistical significance, and n value are reported in the Figure legends. Statistical analyses were performed using Prism Software (GraphPad). For statistical comparison, one-way ANOVA was employed. A value of $p < 0.05$ was considered significant.
