# Supplementary material for: Human embryonic stem cells contribute to embryonic and extraembryonic lineages in mouse embryos upon inhibition of apoptosis
Source: Cell Res. 2017 Nov 3;28(1):126–9. doi: 10.1038/cr.2017.138 (PMC5752840; doi:10.1038/cr.2017.138)
Supplement: Supplementary information, Tables — S1–S2 [file cr2017138x2.pdf]

Table S1. Summary of chimera formation efficiency of the *BCL2L1*- and *BCL2*-overxpression hESCs in pre-implantation mouse embryos.

Donor cell lines	No. of injected embryos	DOX treatment		Chimeric embryos developed *in vitro*	
		Cell culture	Embryo culture	No. of blastocysts (%)	No. of GFP-positive blastocysts (%)
BCL2L1-1	54	+	+	46 (85.2)	46 (100.0)
BCL2L1-2	35	+	+	30 (85.7)	26 (86.7)
BCL2-1	55	+	+	33 (62.3)	30 (90.9)
BCL2-3	62	+	+	53 (85.5)	50 (94.3)
Control-1*	27	+	-	22 (81.5)	12 (54.5)
Control-2*	35	-	-	15 (42.9)	2 (13.3)

* BCL2L1-1 hESCs were used as control.

Table S2. Primers used in the study.

primer name	5'-primer sequence-3'	
NEO-F	AAGATGGATTGCACGCAGGTTCTCC	Identification of virus integration
NEO-R	AAGAAGGCGATAGAAGGCGATGCGC	
BCL2-F	ATCCAGCCTCCGCGGCCCCGGCCACCATGGCGCACGCTGGGAGAACAGG	*BCL2/BCL2L1* overexpression vector construction
BCL2-R	TTTGTTTGACCTTGTGGCCCAGATAGGCACCC	
BCL2-IRES-F	GTGCCTATCTGGGCCACAAGGTCAAACAAACTCTTAACTTTGATT	
BCL2-IRES-R	TATCGATAAGCTTGATATCGAATTTTGTAATCCAGAGGTTGATTGTCG	
BCL2L1-F	GCCTCCGCGGCCCCGGCCACCATGTCTCAGAGCAACCGGGAGCTGG	
BCL2L1-R	TTTCCGACTGAAGAGTGAGCCCAGC	
BCL2L1-IRES-F	CTCTTCAGTCGGAAAGTCAAACAAACTCTTAACTTTGATT	
BCL2L1-IRES-R	ATAAGCTTGATATCGAATTTTGTAATCCAGAGGTTGATTGTCG	
AAVS1-sgRNA-F	CACCGGGGCCACTAGGGACAGGAT	sgRNA sequence
AAVS1-sgRNA-R	AAACATCCTGTCCCTAGTGGCCCC	
hMitochondria-F	AATATTAAACACAAACTACCACCTACCT	Human cell contribution
hMitochondria-R	TGGTTCTCAGGGTTTGTTATAA	

UCNE-F	AACAATGGGTTCAGCTGCTT	assay
UCNE-R	CCCAGGCGTATTTTTGTTCT	
OCT4-F	CTTGAATCCCGAATGGAAAGGG	Real-time PCR
OCT4-R	GTGTATATCCCAGGGTGATCCTC	
NANOG-F	TTTGTGGGCCTGAAGAAAACT	
NANOG-R	AGGGCTGTCCTGAATAAGCAG	
SOX2-F	GCCGAGTGGAAACTTTTGTCG	
SOX2-R	GGCAGCGTGTACTTATCCTTCT	
SOX17-F	GTGGACCGCACGGAATTTG	
SOX17-R	GGAGATTCACACCGGAGTCA	
CXCR4-F	ACTACACCGAGGAAATGGGCT	
CXCR4-R	CCCACAATGCCAGTTAAGAAGA	
T-F	TATGAGCCTCGAATCCACATAGT	
T-R	CCTCGTTCTGATAAGCAGTCAC	
PDGFRA-F	TGGCAGTACCCCATGTCTGAA	
PDGFRA-R	CCAAGACCGTCACAAAAAGGC	

SOX1-F	CAGTACAGCCCCATCTCCAAC	
SOX1-R	GCGGGCAAGTACATGCTGA	
PAX6-F	TGGGCAGGTATTACGAGACTG	
PAX6-R	ACTCCCGCTTATACTGGGCTA	
